# Hybrid Networks of Hyaluronic Acid and Poly(trimethylene carbonate) for Tissue Regeneration

**DOI:** 10.1021/acs.biomac.2c00861

**Published:** 2022-11-23

**Authors:** Anniek M. C. Gielen, Marc Ankone, Dirk W. Grijpma, André A. Poot

**Affiliations:** Department of Advanced Organ Bioengineering and Therapeutics, Faculty of Science and Technology, University of Twente, P.O. Box 217, 7500 AEEnschede, The Netherlands

## Abstract

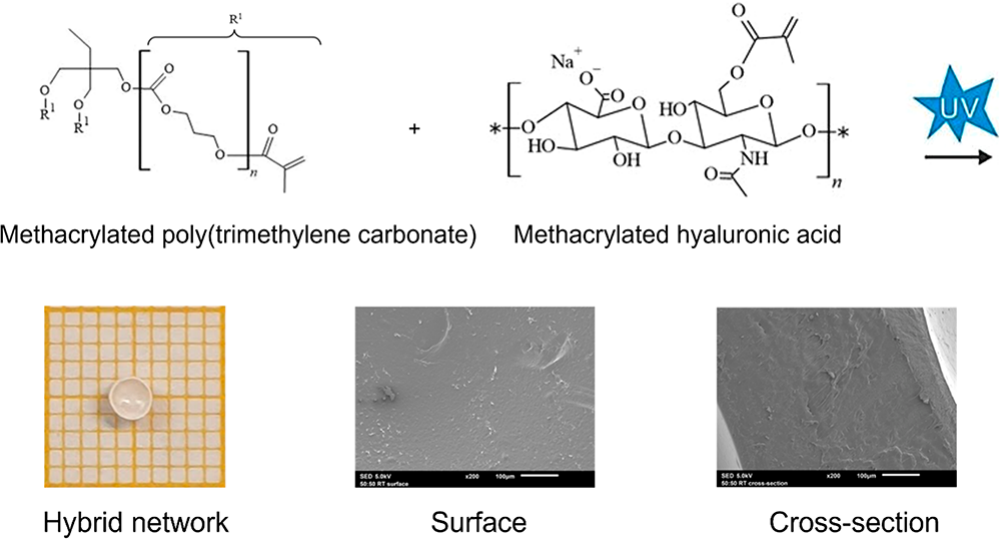

To improve the mechanical performance of hyaluronic acid (HA)-based hydrogels, we prepared novel hybrid hydrogels consisting of hydrophilic HA and hydrophobic poly(trimethylene carbonate) (PTMC). Both polymers were functionalized with methacrylic anhydride, yielding HAMA and PTMC-tMA. Hybrid networks with different ratios of PTMC-tMA:HAMA were prepared by photo-cross-linking, using DMSO pH 2.7 as a common solvent for both macromers. The hybrid networks had high gel contents. The hydrophilicity of the networks increased with increasing HAMA content. The networks consisted of the intended amounts of both macromers. The suture retention strength and compression modulus of the networks increased with increasing PTMC-tMA content. While the 100% HAMA network could not be sutured, the 50:50 PTMC-tMA:HAMA network had a suture retention strength of 5.3 N/mm. This is comparable to that of natural vascular tissues. Also the compression modulus (867 kPa) was significantly higher than that of the 100% HAMA network (13 kPa). Moreover, the networks were compatible with human mesenchymal stem cells. In conclusion, these resilient PTMC-tMA:HAMA networks are promising new biomaterials for tissue regeneration.

## Introduction

Hyaluronic acid (HA) is a natural polysaccharide and an interesting material for biomedical applications because it is biodegradable, biocompatible, and bioresorbable. HA is a major extracellular component of connective tissue and plays a role in the wound healing process. HA is a hydrophilic polymer which is able to maintain a hydrated environment that is favorable for cell infiltration. HA contains functional groups, i.e., the carboxyl and hydroxyl group, that can be used for chemical modification and tailoring of the material properties to the desired application.^1^ HA hydrogels, which can be synthesized by photo-cross-linking methacrylate-functionalized HA, are noncytotoxic and retain intrinsic biological activity.^2^ These networks are effective biomaterials, especially for soft tissue regeneration. However, they are brittle and show poor mechanical performance, degrade rapidly and/or require complicated procedures for their synthesis.^3^ The brittleness of HA hydrogels limits their application in the regeneration of load-bearing tissues.^2^

A strategy to enhance the structural integrity of HA networks is to combine them with a synthetic polymer. Up to now, this has been done with hydrophilic polymers such as poly(dimethyl acrylamide),^2,3^ poly(ethylene glycol),^4,5^ arginine-based poly(ester amide),^6^ and poly(*N*-isopropylacrylamide).^7^ As both HA and the synthetic polymer are hydrophilic, water is used as a common solvent during synthesis of these hybrid networks. Here, we report on the synthesis of hybrid networks consisting of HA and a hydrophobic synthetic polymer, poly(trimethylene carbonate) (PTMC). The rationale of this study was to prepare HA-based hybrid hydrogels with improved resilience, toughness, and handling characteristics as compared to the previously reported HA-based hybrid hydrogels. As in our approach, a hydrophilic and a hydrophobic polymer were used, identification of a common solvent for both polymers was essential for the synthesis of the hybrid networks.

PTMC is a synthetic polymer, synthesized by ring-opening polymerization of trimethylene carbonate (TMC). It is a hydrophobic, biocompatible, biodegradable polymer with excellent mechanical properties, which is dependent on the molar mass.^8,9^ The material undergoes enzymatic surface erosion, which results in longer maintenance of the mechanical integrity as opposed to bulk degradation. Upon cross-linking of PTMC, an elastic network is obtained that can effectively resist creep during long-term cyclic deformation.^10^ Due to the hydrophobic character of PTMC, its bioactivity is relatively low, characterized by unspecific protein adsorption and cell adhesion.^11^

In this study, hybrid networks were developed consisting of HA and PTMC. Both polymers were functionalized using methacrylic anhydride yielding photo-cross-linkable macromers. Dimethyl sulfoxide (DMSO) acidified to pH 2.7 was used as a common solvent for both macromers. It was hypothesized that the hybrid networks would be cytocompatible and that the mechanical performance of the networks would improve by incorporation of PTMC in the networks.

## Materials and Methods

### Materials

HA sodium salt was purchased from Contipro, Czech Republic (molar mass 30–50 kg/mol). TMC was provided by Huizhou Foryou Medical Devices, China. Stannous octoate (Sn(Oct)_2_), triethylamine (TEA), methacrylic anhydride, trimethylol propane (TMP), hydroquinone, 1–4-(2-hydroxyethoxy)-phenyl-2-hydroxy-2-methyl-1-propane-1-one (Irgacure 2959), deuterated chloroform, deuterium oxide, dimethyl sulfoxide, absolute ethanol, hydrochloric acid (HCl), sodium hydroxide (NaOH), and 3 Å molecular sieves (MS) were purchased from Sigma-Aldrich, The Netherlands. Technical grade ethanol was obtained from Boom Chemicals, The Netherlands. Dichloromethane (DCM) was bought from VWR Chemicals, Germany. Dulbecco’s phosphate-buffered saline (DPBS), Dulbecco’s modified Eagle’s medium (DMEM), fetal bovine serum (FBS), glutamax, trypsin/EDTA, and penicillin/streptomycin were obtained from Gibco. Gelatin solution (Type B, 2% (w/v) in water, tissue culture grade), calcein-AM, and ethidium homodimer I were purchased from Sigma-Aldrich.

### HA Functionalization

**Scheme 1:**
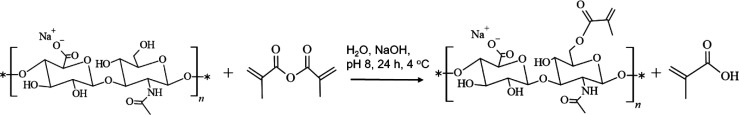
Functionalization of HA with Methacrylic Anhydride in Water, Yielding HAMA with Methacrylic Acid as Byproduct

Methacrylation of HA was performed following the procedure of Oudshoorn et al.^12^ In brief, 8 g of HA sodium salt was dissolved in 400 mL of deionized water (2% (w/v) concentration) and cooled at 4 °C to avoid side reactions. Next, 119.2 mL (40-fold molar excess relative to primary hydroxyl functional group) of methacrylic anhydride was added. The pH of the reaction mixture was kept around 8 with 5 mol/L NaOH and the solution was stirred for 24 h at 4 °C, shielded from light. The solution was precipitated in technical grade ethanol, and the precipitate was washed three times with absolute ethanol and dried in a vacuum oven at approximately 800 mbar at room temperature (RT, ∼20 °C) for 5 d. The degree of functionalization was determined using ^1^H NMR spectral data of methacrylated HA (HAMA) dissolved in D_2_O at a concentration of 5 mg/mL (Scheme 1). The measurements were carried out with a Varian Inova 400 MHz ^1^H NMR spectrometer. The degree of functionalization (DF) of HAMA was calculated according to eq 1:
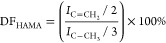
1in which *I*_C=CH_2__ is the integral value of the peaks of the methacrylate vinyl group protons and *I*_C–CH_3__ is the integral value of the peak of the methyl protons of the *N*-acetyl group of HA. Integral values were determined using Mnova 14.3.0 software (Mestrelab Research, Spain).

### PTMC Synthesis and Functionalization

**Scheme 2:**
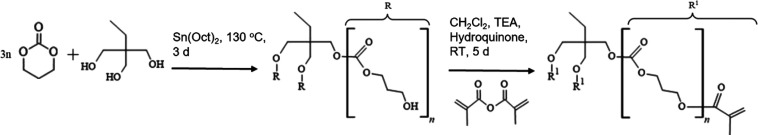
Synthesis of Three-Armed PTMC with TMP as Initiator, Followed by End-Functionalization Using Methacrylic Anhydride with TEA as Catalyst, Yielding Functionalized Three-Armed PTMC (PTMC-tMA)

A three-armed PTMC oligomer was synthesized by ring-opening polymerization of TMC (Scheme 2). The polymerization reaction was performed under inert conditions in the presence of TMP as initiator. The monomer to initiator ratio was adjusted to obtain PTMC oligomer with a molar mass (*M*_n_) of 15 kg/mol. The polymerization was conducted in a three-neck round-bottom flask, where the TMC was heated to 80 °C until it had fully melted, after which the initiator (0.5 mol % relative to TMC) and Sn(Oct)_2_ catalyst (0.13 wt % relative to TMC) were added.^13^ Next, the temperature was increased to 130 °C, and after 3 d, the reaction was stopped by cooling to RT. The end-functionalization was done in dry DCM (dried on MS) under inert conditions, where the hydroxyl end groups reacted with methacrylic anhydride in the presence of TEA as catalyst and hydroquinone as radical scavenger. First, 15 g of PTMC was dissolved in 68.5 mL of dry DCM (4.5 mL/g oligomer), followed by the addition of 15 mg of hydroquinone (0.1 wt % relative to oligomer), 1.25 mL of TEA (9 mol/mol oligomer), and 1.33 mL of methacrylic anhydride (9 mol/mol oligomer). The reaction was conducted for 5 d at RT under continuous stirring in the dark.^13^ The PTMC-tMA macromer was purified by precipitation in cold ethanol and dried in a vacuum oven at slightly elevated temperature (50–55 °C) in the dark. The *M*_n_ of PTMC and DF of PTMC-tMA were determined using ^1^H NMR spectral data of the polymer and macromer dissolved in chloroform-*d* at a concentration of 5 mg/mL.^13^ The *M*_n_ was calculated according to eq 2:
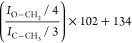
2where *I*_O–CH_2__ is the integral value of the PTMC O–CH_2_ protons, *I*_C–CH_3__ is the integral value of the TMP initiator CH_3_ protons, 102 is the molar mass of the TMC repeating unit, and 134 is the molar mass of TMP. The DF was calculated according to eq 3:
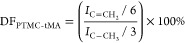
3where *I*_C=CH_2__ is the integral value of the peaks of the methacrylate vinyl group protons and *I*_C–CH_3__ is the integral value of the peak of the TMP methyl protons.

### Preparation of Photo-Cross-Linked Networks

PTMC-tMA and HAMA were separately dissolved in DMSO pH 2.7 (970 μL HCl was added to 1L DMSO) at a concentration of 10% (w/v) at RT. Irgacure 2959 was added as a photoinitiator, for HAMA 1 wt % and for PTMC-tMA 0.1 wt % relative to the macromer. Five different mixed macromer networks were prepared from mixtures with PTMC-tMA:HAMA ratios of 100:0, 75:25, 50:50, 25:75, and 0:100 (w/w), respectively. The mixtures were poured into a mold, placed between two quartz glass plates, and photo-cross-linked by irradiation for 60 min at 365 nm in a UV box.

### Characterization of the Photo-Cross-Linked Networks

#### Sol–Gel Extraction

After photo-cross-linking, the networks were swollen to equilibrium in DMSO pH 2.7. This allows for the extraction of the soluble (sol) fraction of the cross-linked networks. The initial mass of the networks just after cross-linking was recorded (*m*_0_), before they were placed in DMSO pH 2.7. The networks were swollen for 48 h and weighed after gently blotting excess solvent with Kimwipes. Next, free reagents and solvent were extracted by continuously adding deionized water to the swollen networks at a rate of 1 mL/min for 32 h, while keeping the volume constant. Subsequently, the networks were immersed in 100% deionized water and refreshed regularly for another 24 h. Finally, the networks were freeze-dried to determine their dry mass (*m*_dry_). The percentage gel content was determined according to eq 4:
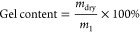
4where
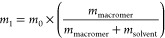


#### Water Uptake

Water uptake analysis was done to determine the capacity of the networks to retain water. The extracted and dried networks (*m*_dry_) were swollen in deionized water at RT for 24 h. The swollen networks were gently blotted with Kimwipes to remove excess liquid and weighed (*m*_swollen_). The water uptake was determined according to eq 5:
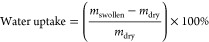
5

#### Water Contact Angle

Water contact angles were determined by the captive bubble technique, using an optical contact angle measuring device. The network was swollen in deionized water for 24 h and placed in water against a substrate. A syringe needle was positioned in the water underneath the network and an air bubble was placed against the surface, after which the contact angle was measured.

#### Scanning Electron Microscopy

The surface and cross-section morphologies of the networks were examined by scanning electron microscopy (SEM), using a JEOL JSM-IT100 at 5.0 kV. For the cross-section samples, the extracted and dried networks were cut in half by first immersing them in liquid nitrogen, followed by cutting them with one movement using a blade and hammer. This method was used to avoid pressing the material together, minimizing the possibility to alter the morphology. Gold sputtering was done in a Cressington Sputter Coater 108 auto with a pure gold target at 10 mA for 60 s.

#### Differential Scanning Calorimetry

Differential scanning calorimetry (DSC) was carried out using a TA Instruments DSC25. A piece of 15 mg dry sample was sealed in an aluminum DSC pan, an empty DSC pan was used as a reference. The temperature range was set between −110 and 35 °C, with a heating and cooling rate of 10 °C/min. The glass transition temperature (*T*_g_) was determined as the midpoint value of the heat capacity change of the second heating scan.

#### Attenuated Total Reflectance-Fourier Transform Infrared Spectroscopy

Dry samples were placed against the attenuated total reflectance (ATR) crystal of a Bruker Alpha-P. The resolution of the machine was 4 cm^–1^, 32 scans were made, and the data between 4000 and 400 cm^–1^ were saved. A background scan was made with an empty crystal and subtracted from every sample measurement. Data analysis was done using OPUS software.

#### Compression Testing and Suture Retention Strength

Compressive stress–strain measurements were carried out using a TA Instruments DMA 850. Networks were photo-cross-linked in a disk shape of 6 mm diameter and 3 mm thickness, extracted, and freeze-dried. Next, they were swollen in water for 5 d. Samples were transferred to the DMA, allowed to equilibrate for 5 min at 20 °C and measured in the hydrated state. A preload of 0.01 g was applied before starting a measurement, which was done at a deformation rate of 0.1 mm/min. The compression modulus (*E*_c_) was taken as the slope of the stress–strain curve in the linear area below 50% strain.

Suture retention strength (SRS) of the networks was determined using hydrated samples with an approximate thickness and width of 0.5 mm and 3 mm, respectively. At a distance of 2 mm from the top of a sample, a hole was punctured with a syringe needle (0.5 × 25 mm/25G × 1 in., Terumo, Belgium). After inserting a stainless steel wire (Monacor, Germany) with a diameter of 0.1 mm through this hole, both ends of the wire were clamped in the upper grip of the DMA 850 operating in tensile mode. The other side of the sample was clamped in the lower grip, after which the force required to elongate and tear the sample was determined at a crosshead speed of 0.5 mm/min. The value for the SRS was normalized to the sample thickness and given in N/mm.

### Cell Culturing

Human mesenchymal stem cells (hMSCs, passage 5) were cultured at 37 °C in humidified air containing 5 vol % CO_2_, in 75 cm^2^ cell culture flasks containing culture medium consisting of DMEM, 1% (v/v) glutamax, 10% (v/v) FBS, and 1% (v/v) penicillin/streptomycin. The culture flasks were coated with 0.1% (v/v) gelatin solution in sterile water before cell seeding. The medium was refreshed three times per week until the cells reached confluence. Upon confluence, the cells were trypsinized and counted using an EVE automated cell counter. The 5 different macromer networks were cut to disk-shaped samples with a diameter of 10 mm and a thickness of 1 mm and placed in a 48 wells suspension culture plate (not surface-treated for cell culturing). Silicone O-rings (Technirub, The Netherlands) with 11.3 mm outer and 9.52 mm inner diameter were extracted with absolute ethanol and put on top of the networks to prevent them from floating. Subsequently, the networks and rings were disinfected with 70% (v/v) ethanol in water for 10 min, washed with DPBS, and kept in cell culture medium overnight at 37 °C. The hMSCs were seeded on the networks at a density of 8000 cells per well and cultured for 7 d. The medium was refreshed three times per week. Live/dead staining was performed on d 7 after cell seeding. The networks were rinsed with warm DPBS (37 °C) and incubated with 2 μM calcein-AM/4 μM ethidium homodimer-1 solution in culture medium for 1 h. After rinsing with warm DPBS, pictures were taken using an EVOS FL cell imaging system.

### Statistical Analysis

Differences between outcomes of analyses using multiple networks were evaluated by one-way ANOVA using Bonferoni posthoc analysis (IBM SPSS Statistics 28) and considered statistically significant when *p* < 0.05.

## Results and Discussion

### Synthesis and Characterization of Functionalized HA

HA (molar mass 30–50 kg/mol) was functionalized in water at pH ∼ 8 with a 40-fold excess of methacrylic anhydride relative to its primary hydroxyl group. The DF of the synthesized macromer was determined by ^1^H NMR spectroscopy. Spectral data showed that the DF of HAMA was influenced by the excess of methacrylic anhydride as well as the pH during the reaction (see Figure S1).

**Figure 1:**
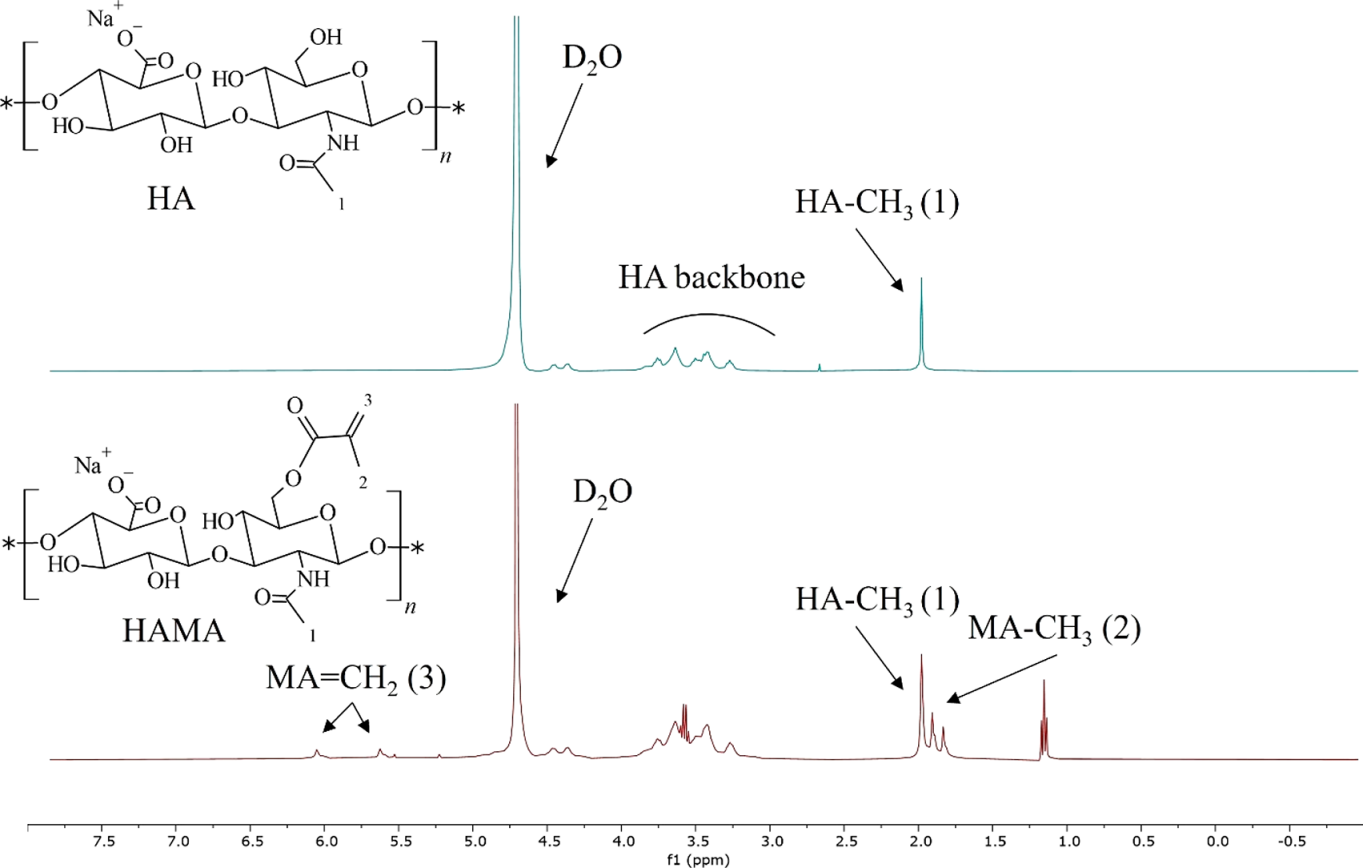
^1^H NMR spectra of HA (top) and HAMA (bottom) dissolved in D_2_O.

For the preparation of the hybrid networks, HAMA with a DF of 39% was used, as determined by ^1^H NMR analysis (see Figure 1). The spectra confirmed the presence of methacrylate vinyl group protons (C=CH_2_) at δ 5.6 and 6.1 ppm, indicating the conversion of HA to HAMA. The DF was calculated according to eq 1, using the integral values of the peaks of the methacrylate vinyl group protons (3) and the peak of the CH_3_ protons of the *N*-acetyl group of HA (1) at δ 2.0 ppm.^14^ The integral values are shown in Table S1. The ratio of the integral values of the methacrylate vinyl group protons and the methacrylate methyl group protons ((3) and (2) in Figure 1, respectively)^14^ was 2:3, which is in agreement with the ratio of these protons in the methacrylate group. The peaks at δ 1.17 and 3.65 ppm originated from ethanol that was still present after the drying process.

### Synthesis and Characterization of PTMC

**Figure 2:**
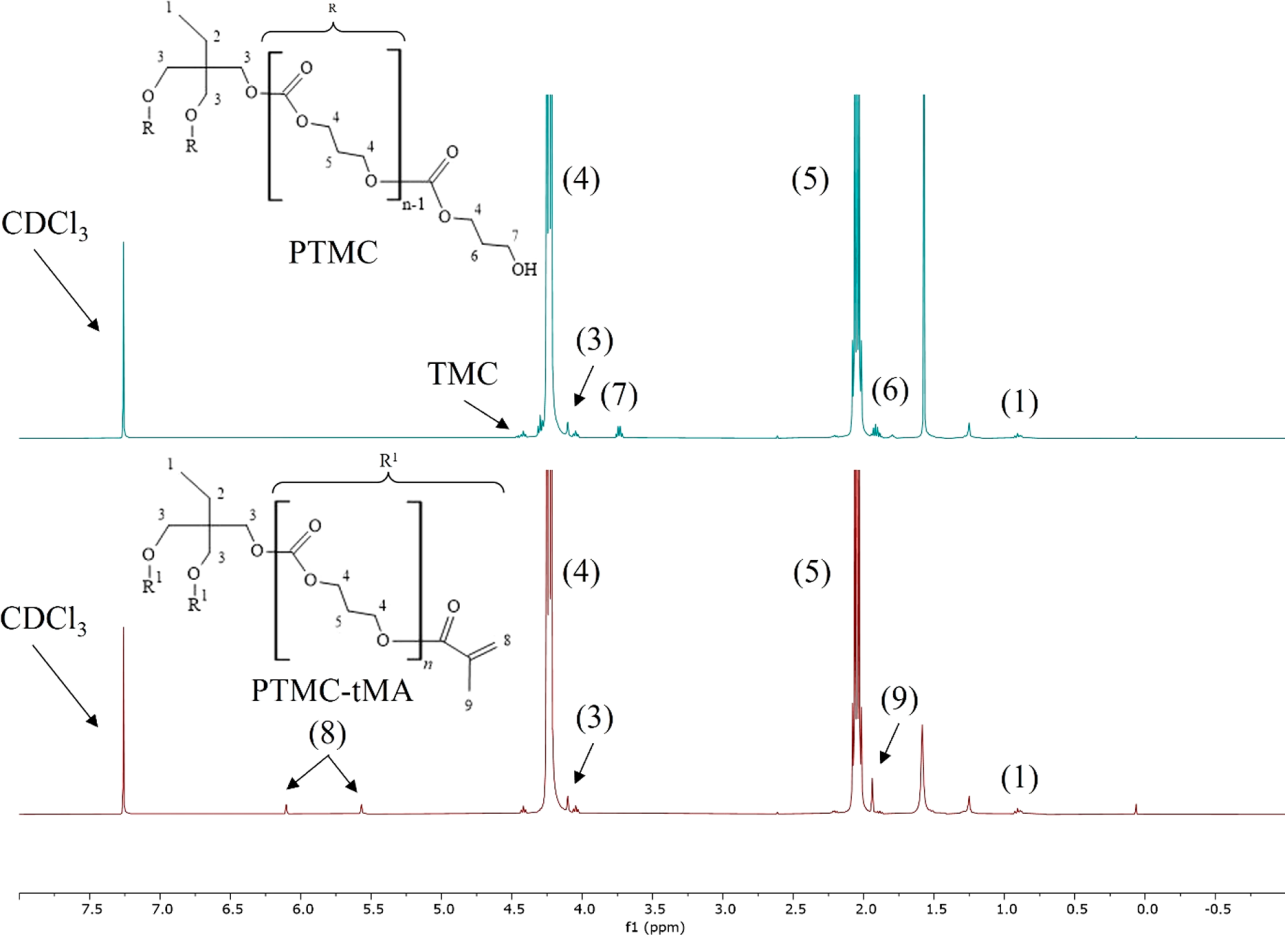
^1^H NMR graphs of PTMC (top) and PTMC-tMA (bottom) dissolved at a concentration of 5 mg/mL in CDCl_3_.

Three-armed PTMC oligomer was synthesized by ring-opening polymerization of TMC with TMP as initiator. By comparing the integral value of the TMP CH_3_ peak at δ 0.91 ppm (1) with the integral value of the PTMC methylene peak at δ 4.24 ppm (4), an *M*_n_ of 15.3 kg/mol was calculated (see eq 2 and Figure 2, top graph).^13^ The integral values are shown in Table S1. The choice for PTMC with a molar mass of 15 kg/mol was based on previous work regarding the preparation of PTMC networks^9^ as well as PTMC/Gelatin hybrid networks.^15^ The polymer was functionalized with methacrylic anhydride in dry DCM for 5 d, resulting in PTMC-tMA. The presence of methacrylate groups was confirmed by the peaks of the vinyl group protons at δ 6.11 and 5.57 ppm (8) (see Figure 2, bottom graph).^13^ The DF was calculated according to eq 3, using the integral values of these peaks and that of the CH_3_ peak of the initiator at δ 0.91 ppm (1). The integral values are shown in Table S1. The reaction resulted in PTMC-tMA with a DF of 90%. In both spectra shown in Figure 2, there are some peaks that do not correspond with PTMC or PTMC-tMA. The peak at δ 1.56 ppm can be attributed to H_2_O. The peaks at δ 0.8 and 1.25 ppm originated from the vacuum grease used during synthesis of the polymer.

### PTMC-tMA:HAMA Mixtures and Networks

**Figure 3:**
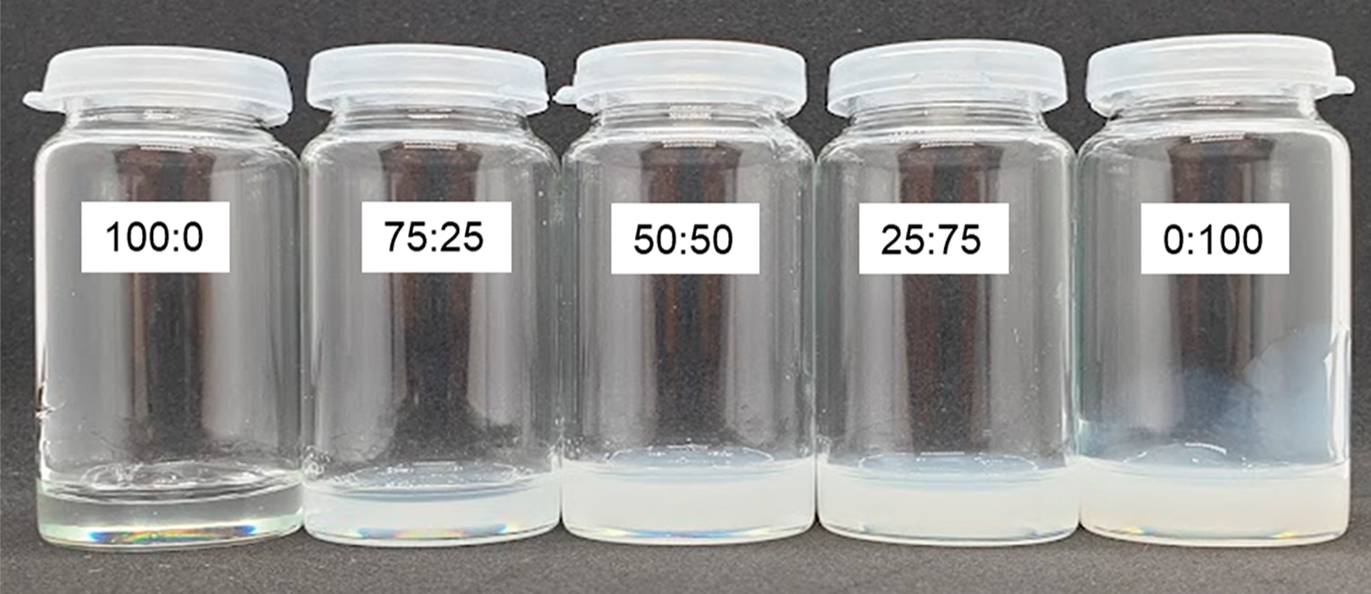
Macromer mixtures with different PTMC-tMA:HAMA ratios in DMSO pH 2.7 at a concentration of 10% (w/v).

**Figure 4:**
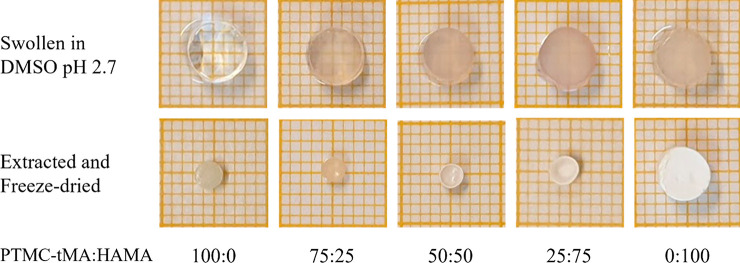
Macroscopic appearance of PTMC-tMA:HAMA networks swollen in DMSO pH 2.7 after cross-linking and after subsequent extraction and freeze-drying. Small squares are 1 × 1 mm.

Hybrid networks with different ratios of PTMC-tMA:HAMA were synthesized. First, both polymers were dissolved separately at a concentration of 10% (w/v) in DMSO pH 2.7 and photoinitiator was added. Next, they were mixed at different (w/w) ratios of 100:0, 75:25, 50:50, 25:75 and 0:100. As shown in Figure 3, PTMC-tMA in DMSO pH 2.7 was a clear and transparent solution, whereas HAMA in DMSO pH 2.7 yielded a homogeneous but turbid system. Next, the mixtures were photo-cross-linked, extracted, and lyophilized, see Figure 4. Upon irradiation of methacrylate-functionalized polymers in the presence of photoinitiator, the methacrylate groups polymerize via an addition-type reaction forming polymethacrylate cross-links, and thus a network is formed.^16,17^

### Physical Properties of PTMC-tMA:HAMA Networks

The gel contents of all networks were higher than 82%, indicating efficient cross-linking (data not shown). The water uptake of the networks increased with increasing HAMA content, see Table 1. This can be attributed to the hydrophilic nature of HAMA. Water uptake is an important property in tissue engineering, as it allows for a hydrated environment that facilitates the supply of nutrients.

**Table 1:** Properties of Networks with Different PTMC-tMA:HAMA Ratios, Photo-Cross-Linked at a 10% (w/v) Macromer Concentration^a^

PTMC-tMA: HAMA	WU (%)	Water Contact Angle (deg)	*T*_g_ (°C)
100:0	9.4 ± 0.1	134.6 ± 0.9	–15.0
75:25	44.2 ± 2.1	67.2 ± 1.4	–14.4
50:50	105.9 ± 0.9	41.7 ± 4.2	–14.9
25:75	205.6 ± 6.6	30.9 ± 1.3	–15.4
0:100	717.6 ± 41.3	–	–

a WU = Water uptake; *T*_g_ = glass transition temperature. All values *n* = 3, except *T*_g_*n* = 1.

In line with the water uptake results, the water contact angle measurements indicate an increase of hydrophilicity with a larger proportion of HAMA in the networks. The water contact angle decreased with increasing HAMA content, indicating an increase in wettability of the surface. The water contact angle could not be recorded for the 100% HAMA network, as the surface was too hydrophilic. The air bubble would not easily leave the needle, and once it detached, it immediately rolled off the 100% HAMA surface. This indicates that the water contact angle for the 100% HAMA network was close to 0°.

A *T*_g_ for the 100% HAMA network could not be found. For the reference materials, unfunctionalized HA and functionalized un-cross-linked HA, also no *T*_g_ could be observed between −110 and 35 °C. The *T*_g_ of HA is mostly found in the hydrated state,^18,19^ whereas the samples in the present study were analyzed in the dry form. Unfunctionalized PTMC and functionalized un-cross-linked PTMC had a *T*_g_ of −18.2 °C and −23.4 °C, respectively, which is in agreement with previous research of our group.^20^ For the hybrid networks and the 100% PTMC-tMA network, a single *T*_g_ was found. The presence of a single *T*_g_ is commonly used as an indicator to reflect the miscibility of polymers, whereas immiscible polymers show multiple *T*_g_s.^21^ Since no *T*_g_ could be found for HA and HAMA, we cannot conclude about the miscibility of the polymers. The *T*_g_ of the networks was higher than that of unfunctionalized PTMC and PTMC-tMA, which indicates that cross-linked networks were produced. Cross-linking decreases free volume, which is related to a higher *T*_g_.

**Figure 5:**
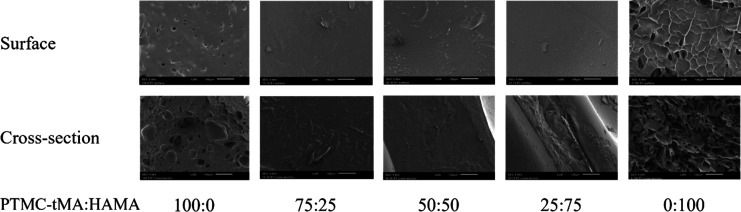
SEM images of surface and cross-section of PTMC-tMA:HAMA networks, photo-cross-linked at a 10% (w/v) macromer concentration. Magnification, 200×; scale bar, 100 μm.

The morphology of the surface and cross-section of the dry networks can be observed in Figure 5. Whereas the hybrid networks were nonporous, the 100% PTMC-tMA and 100% HAMA networks were slightly porous. The pores of the 100% PTMC-tMA network could be attributed to traces of DMSO entrapped in the network during extraction. The porosity of the 100% HAMA network could be explained by the homogeneous but phase-separated system in the solvent DMSO pH 2.7.

**Figure 6:**
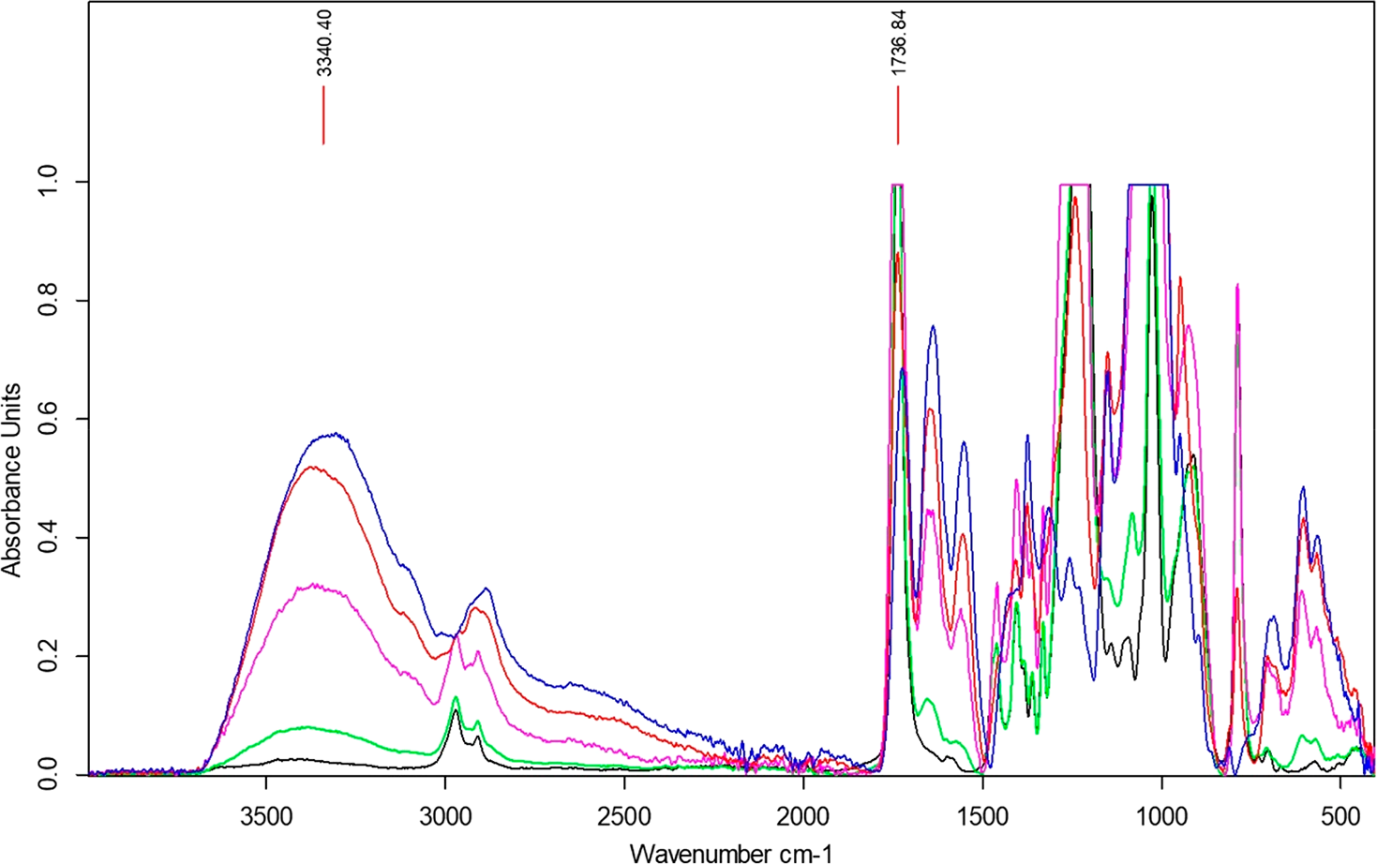
ATR-FTIR spectra of PTMC-tMA:HAMA networks. Black = 100:0; green = 75:25; pink = 50:50; red = 25:75; blue = 0:100 PTMC-tMA:HAMA.

The final polymer ratios after extraction of the hybrid networks were investigated using attenuated total reflectance-Fourier transform infrared spectroscopy (ATR-FTIR) analysis. By comparing characteristic peaks of both polymers, the presence of each could be semiquantified. Figures S2 and S3 show the ATR-FTIR spectra of the initial materials with identification of the characteristic peaks. The broad peak around 3340 cm^–1^ was used for HAMA and the sharp peak at 1736 cm^–1^ for PTMC-tMA, see Figure 6.

**Figure 7:**
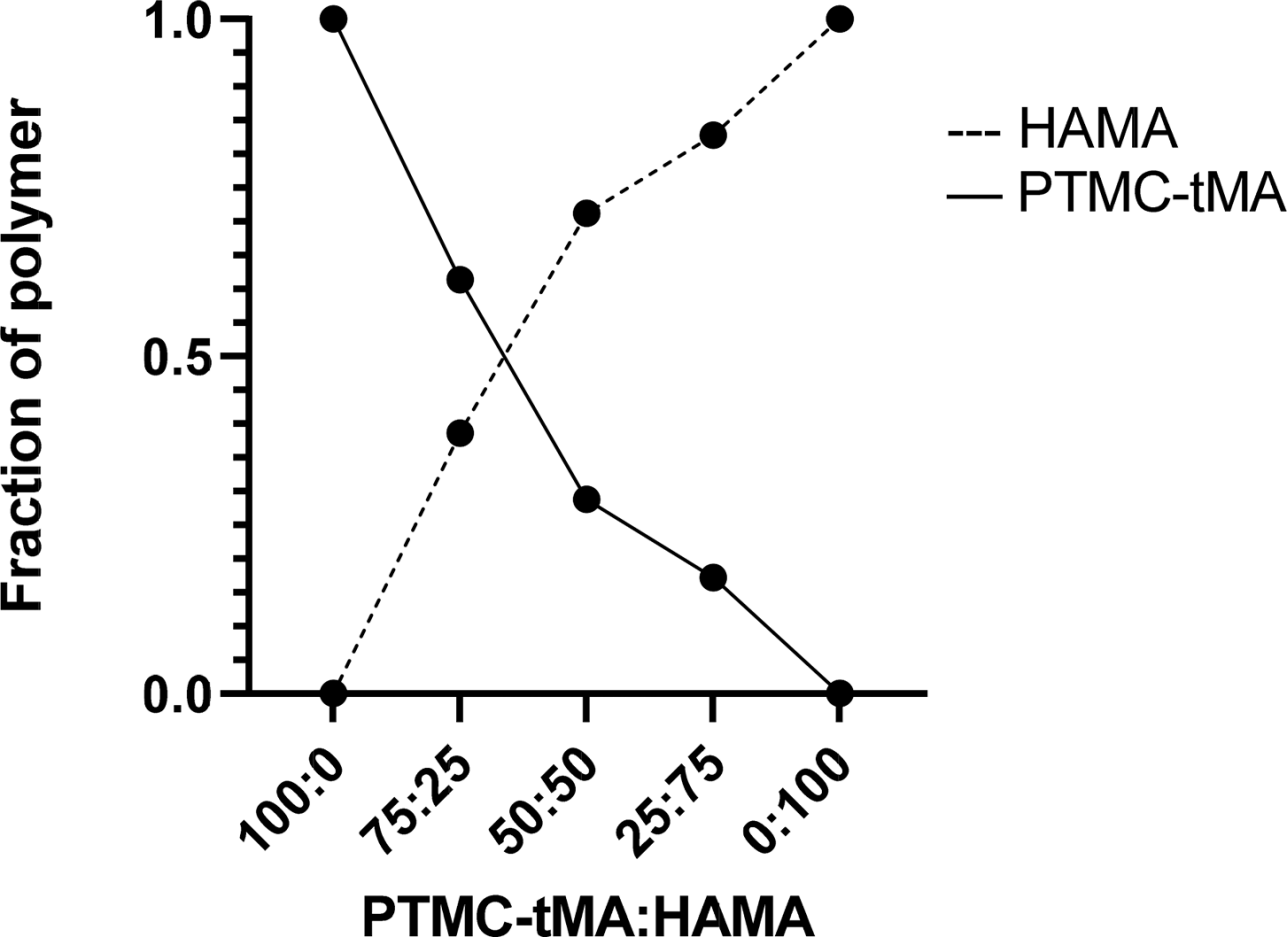
Polymer fractions in the hybrid networks calculated using the ATR-FTIR spectra. Dotted line is HAMA fraction, and solid line is PTMC-tMA fraction.

To estimate the fraction of both polymers in the hybrid networks, the areas under the characteristic peaks of HAMA and PTMC-tMA were determined. This semiquantification shows that both macromers were present in the hybrid networks, approximately in the ratios aimed for, see Figure 7.

Mechanical properties of the networks were determined by compression testing and by measuring the suture retention strength, see Table 2. The networks were photo-cross-linked, extracted, freeze-dried, and swollen in water for 5 d.

**Table 2:** Mechanical Properties of Hydrated PTMC-tMA:HAMA Networks^a^

PTMC-tMA: HAMA	SRS (N/mm)	*E*_c_ (kPa)
100:0	2.3 ± 0.3^b^	4860 ± 677
75:25	3.5 ± 0.3	2213 ± 857^e^
50:50	5.3 ± 0.1^d^	867 ± 25
25:75	1.1 ± 0.0	517 ± 105
0:100	–c	13 ± 6

a SRS = Suture retention strength, *E*_c_ = Compression modulus, all values *n* = 3.

b Samples did not tear.

c Could not be determined as samples were too fragile.

d *p* < 0.05 compared to the SRS of all other networks.

e *p* < 0.05 compared to the *E*_c_ of all other networks.

For tissue engineering applications, it is of utmost importance that engineered structures can be sutured. The toughness of the 100% HAMA network could not be assessed quantitively, as the brittleness of the samples made it impossible to carry out suture retention measurements. However, when PTMC was incorporated in the network, the SRS increased, reaching a value of 5.3 N/mm for the 50:50 PTMC-tMA:HAMA hybrid network. This value was significantly higher compared to the other hybrid networks as well as the 100% PTMC-tMA network. The samples of the latter network, however, did not tear during the measurements. Thus, the maximum SRS of the 100% PTMC-tMA network was probably higher than 2.3 ± 0.3 N/mm, which is in agreement with data previously reported by us (approximately 5 N/mm using PTMC-tMA of 10–20 kg/mol).^9^ In that study, we also determined the SRS of porcine aorta and carotid artery (2.2 and 5.8 N/mm, respectively),^9^ which corresponds to values reported for human femoral artery (2 N/mm)^22^ and human saphenous vein and internal mammary artery (3.6 and 2.5 N/mm, respectively).^23^ Thus, the SRS value of 5.3 N/mm for the 50:50 PTMC-tMA:HAMA network indicates the good suturability of this network to vascular tissues.

The 100% HAMA network had a compression modulus of 13 kPa, which corresponds to data reported in literature.^24−26^ For the hybrid networks, the moduli increased with increasing PTMC-tMA content up to 2.2 MPa in case of the 75:25 PTMC-tMA:HAMA network. This value was significantly higher compared to the other hybrid networks as well as the 100% HAMA network. The relatively high compression moduli of the 75:25 and 50:50 PTMC-tMA:HAMA networks (2.2 and 0.9 MPa, respectively) contribute to their good handling characteristics. Moreover, these values are substantially higher than reported for HA/PEG and HA/arginine-based poly(ester amide) hybrid networks (0.2 and 0.1 MPa, respectively)^4,6^ as well as for a HA/poly(dimethyl acrylamide) interpenetrating network (0.5 MPa).^2^ A compression modulus of approximately 5 MPa for the 100% PTMC-tMA network is in agreement with previous work of our group.^9^ Representative stress–strain curves of the five different networks are shown in Figure S4.

**Figure 8:**
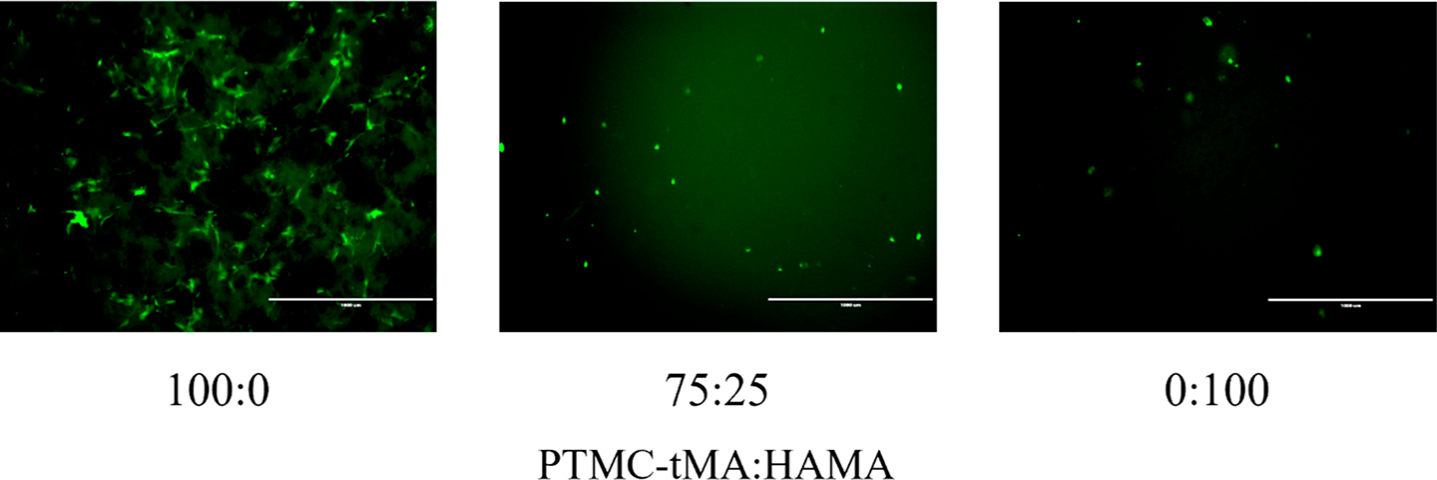
Live/dead staining (green/red, respectively) of hMSCs cultured for 7 d on some of the networks. Magnification, 4×; scale bar, 1000 μm.

Figure 8 shows live/dead staining of hMSCs cultured for 7 d on the 100:0, 75:25 and 0:100 PTMC-tMA:HAMA networks. No dead cells were observed. The 100% PTMC-tMA network contained a relatively large number of cells which had spread on the surface. In contrast, the 75:25 hybrid network and the 100% HAMA network contained less cells which had a round shape. The latter observation corresponds to literature data about the culturing of hMSCs in cross-linked HA hydrogels.^25−28^ The round morphology of the cells was attributed to the low cell adhesive properties of HA and the high cross-link densities of the gels prohibiting cell spreading. Indeed, HA hydrogels with Arg-Gly-Asp cell adhesion motifs and matrix metalloprotease-sensitive degradation sites showed spreading of hMSCs inside the gels.^25−28^

As we used DMSO pH 2.7 as a common solvent, we could not include the cells during preparation of the hybrid hydrogels, but seeded the cells on top of the networks after extraction. Because the cells did not spread, except on the 100% PTMC-tMA network, a substantial number of cells was lost during refreshments of the culture media. This explains the low cell numbers on the HA-based hydrogels in Figure 8. Cell seeding inside the hybrid hydrogels presented in this study would be possible using porous structures. These can easily be made by freezing the PTMC-tMA:HAMA mixtures before and during photo-cross-linking. Below a temperature of approximately 10 °C, DMSO crystallizes and acts as a pore former during photo-cross-linking and subsequent extraction. Examples of porous PTMC-tMA:HAMA hybrid networks are shown in Figure S5. Thus, the use of DMSO pH 2.7 as a common solvent for PTMC-tMA and HAMA will most likely not prohibit cell culturing inside PTMC-tMA:HAMA hybrid hydrogels.

## Conclusions

In order to improve the mechanical performance of HA-based hydrogels, we prepared novel tough hybrid hydrogels consisting of hydrophilic HA and hydrophobic PTMC. This is important as HA hydrogels themselves are brittle, cannot be sutured, and are not suited for application in the regeneration of load-bearing tissues. The preparation of HA-based hybrid hydrogels containing a synthetic hydrophobic polymer has not been reported before. DMSO pH 2.7 was used as a common solvent, yielding a transparent solution of PTMC-tMA and a homogeneous but turbid system in the case of HAMA. Mixtures of different ratios of the functionalized polymers were successfully photo-cross-linked, resulting in hybrid networks with different properties. Increasing the HAMA content of the hybrid networks resulted in a lower water contact angle of the surface and a higher water uptake by the network. The hybrid networks consisted of both macromers in the intended ratios. For the 50:50 PTMC-tMA:HAMA network, the compression modulus (867 kPa) increased 6569% compared to that of the 100% HAMA network (13 kPa). With respect to the suture retention strength (5.3 N/mm for the hybrid hydrogel), such an increase could not be calculated as the brittle 100% HAMA hydrogel immediately failed under tension. Moreover, the networks were compatible with human mesenchymal stem cells. In conclusion, these tough and resilient PTMC-tMA:HAMA hybrid networks are promising new biomaterials for tissue engineering applications.

## Supporting Information Available

Figure S1: ^1^H NMR spectra showing effect of MA concentration on degree of HA functionalization. Figure S2: ATR-FTIR spectra of HA and HAMA. Figure S3: ATR-FTIR spectra of PTMC and PTMC-tMA. Figure S4: Compression stress–strain curves of PTMC-tMA:HAMA networks. Figure S5: Macroscopic appearance of porous PTMC-tMA:HAMA networks (PDF)
