# Supplementary material for: Hybrid Networks of Hyaluronic Acid and Poly(trimethylene carbonate) for Tissue Regeneration
Source: Biomacromolecules. 2022 Nov 23;24(10):4366–74. doi: 10.1021/acs.biomac.2c00861 (PMC10565833; doi:10.1021/acs.biomac.2c00861)
Supplement: Supplementary file 1 — bm2c00861_si_001.pdf [file bm2c00861_si_001.pdf]

Hybrid Networks of Hyaluronic Acid and Poly(trimethylene carbonate) for Tissue Regeneration

Anniek M. C. Gielen, Marc Ankone, Dirk W. Grijpma, André A. Poot*

Department of Advanced Organ Bioengineering and Therapeutics, Faculty of Science and Technology, University of Twente, P.O. Box 217, 7500 AE Enschede, The Netherlands

*Corresponding author, a.a.poot@utwente.nl

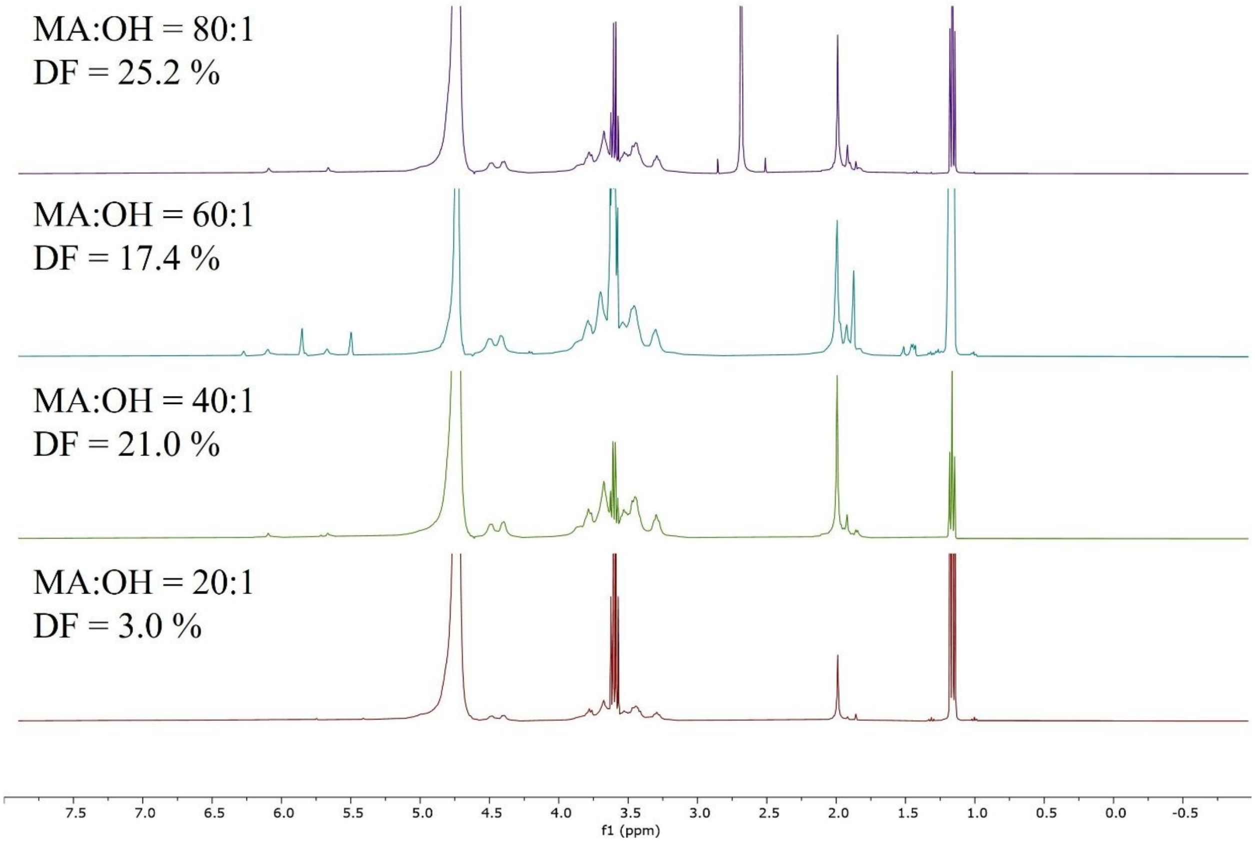

Figure S1. ^{1}H-NMR spectra of HA functionalized with different amounts of molar excess of methacrylic anhydride (MA) compared to the primary hydroxyl group of HA. The degree of functionalization (DF) was calculated using equation 1. The peaks at δ 5.6 and 6.1 ppm correspond to the C=CH_2 group of MA and the peak at δ 2.0 ppm corresponds to the CH_3 group of HA. The DF increased with increasing ratio of MA:OH, except for the 40:1 and 60:1 ratios. This was probably due to lack of control of the pH during the reaction.

Table S1. Integral values of proton peaks from ^{1}H-NMR spectra used for calculation of the degree of functionalization (DF) of HAMA, the number average molar mass (M_n) of PTMC and the DF of PTMC-tMA.

**Calculation**	**Protons**	**δ (ppm)**	**Integral value**
DF HAMA	$C{=}CH_2$	5.6	0.14
	$C{=}CH_2$	6.1	0.12
	$C\text{-}CH_3$	2.0	1
Ratio of CH_2 and CH_3 protons in methacrylate group	$C{=}CH_2$	5.6	0.14
	$C{=}CH_2$	6.1	0.12
	$C\text{-}CH_3$	1.9	0.39
M_n PTMC	$O\text{-}CH_2$	4.24	198.25
	$C\text{-}CH_3$	0.91	1
DF PTMC-tMA	$C{=}CH_2$	5.57	0.92
	$C{=}CH_2$	6.11	0.89
	$C\text{-}CH_3$	0.91	1

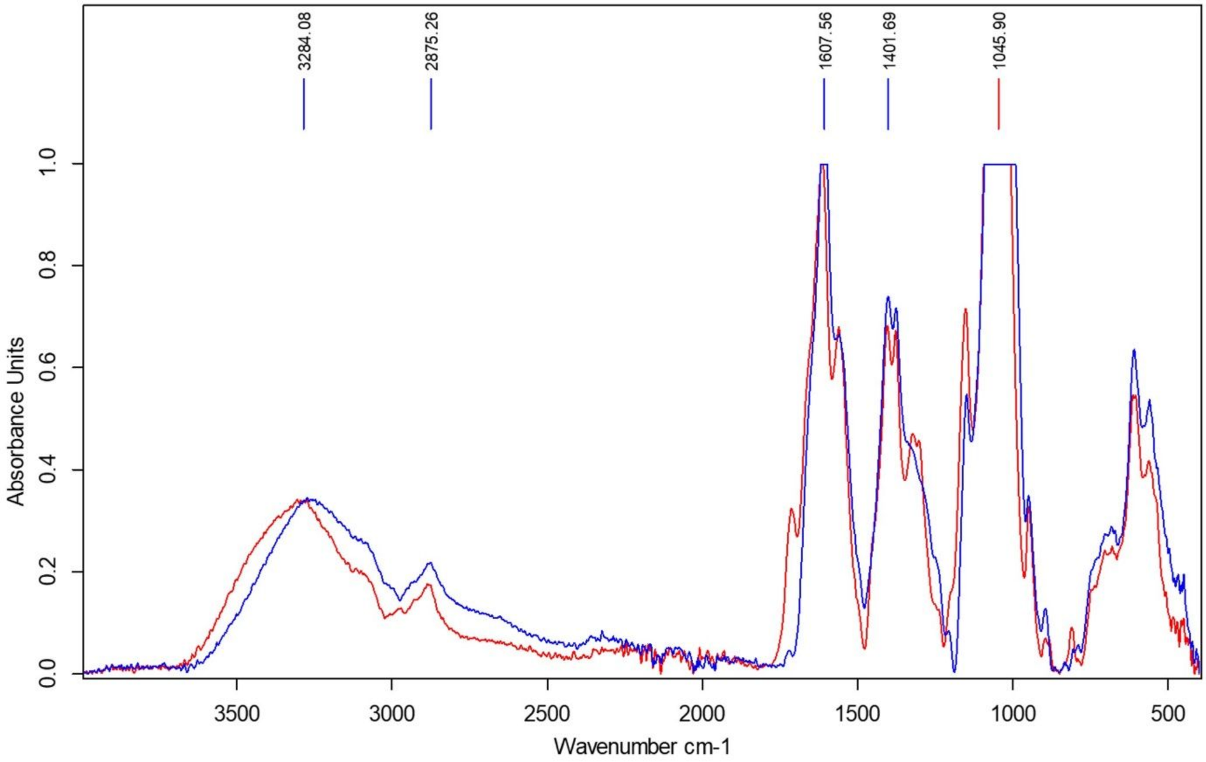

Figure S2. ATR-FTIR spectra of HA (blue) and HAMA (red). The broad signal around 3284 cm^{-1} is associated with intra- and intermolecular stretching vibration of OH and NH groups. The peak at 2875 cm^{-1} corresponds to symmetric stretching vibrations of the CH_2 group. Asymmetric vibrations of the COO^- group can be correlated with the peaks at 1607 cm^{-1} and 1401 cm^{-1}. The C-O-C hemiacetalic system of the saccharide units generates a peak at 1045 cm^{-1} [1, 2].

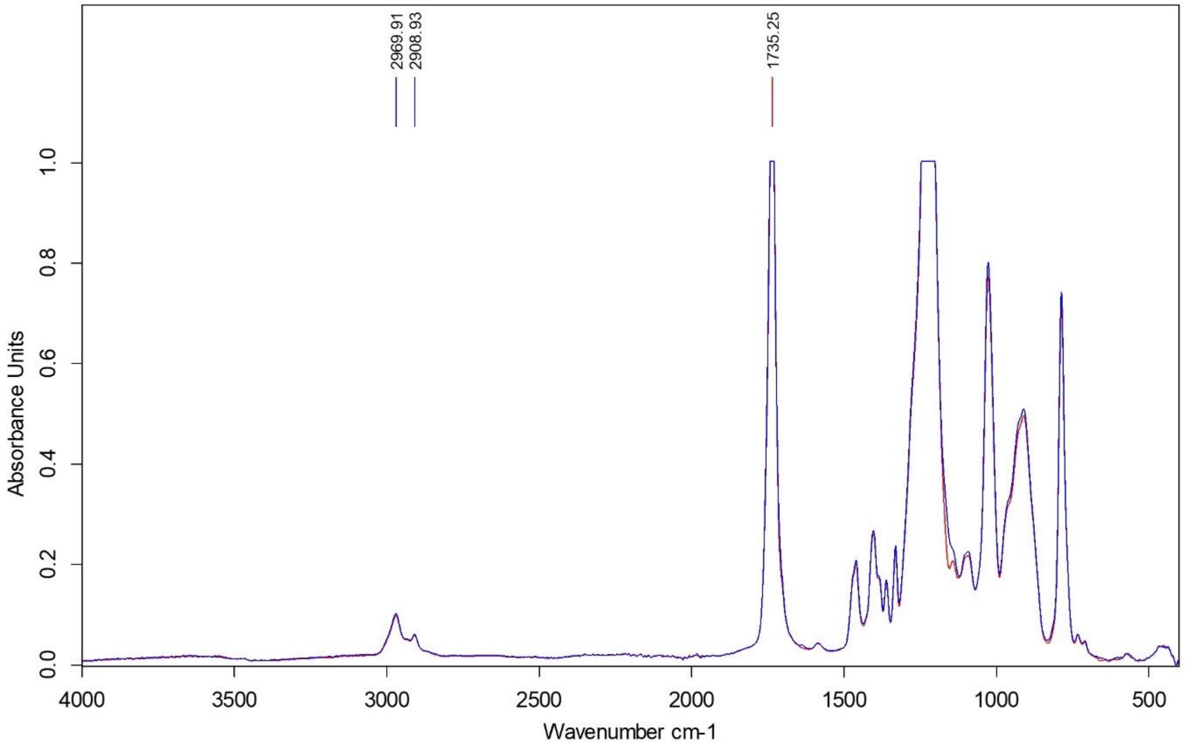

Figure S3. ATR-FTIR spectra of PTMC (red) and PTMC-tMA (blue). The spectral fingerprint of PTMC is observed between 800 and 1500 cm^{-1}. The peak at 1735 cm^{-1} corresponds to stretching vibrations of the C=O group, and the peaks at 2969 cm^{-1} and 2908 cm^{-1} to stretching vibrations of the CH_2 group[3, 4, 5].

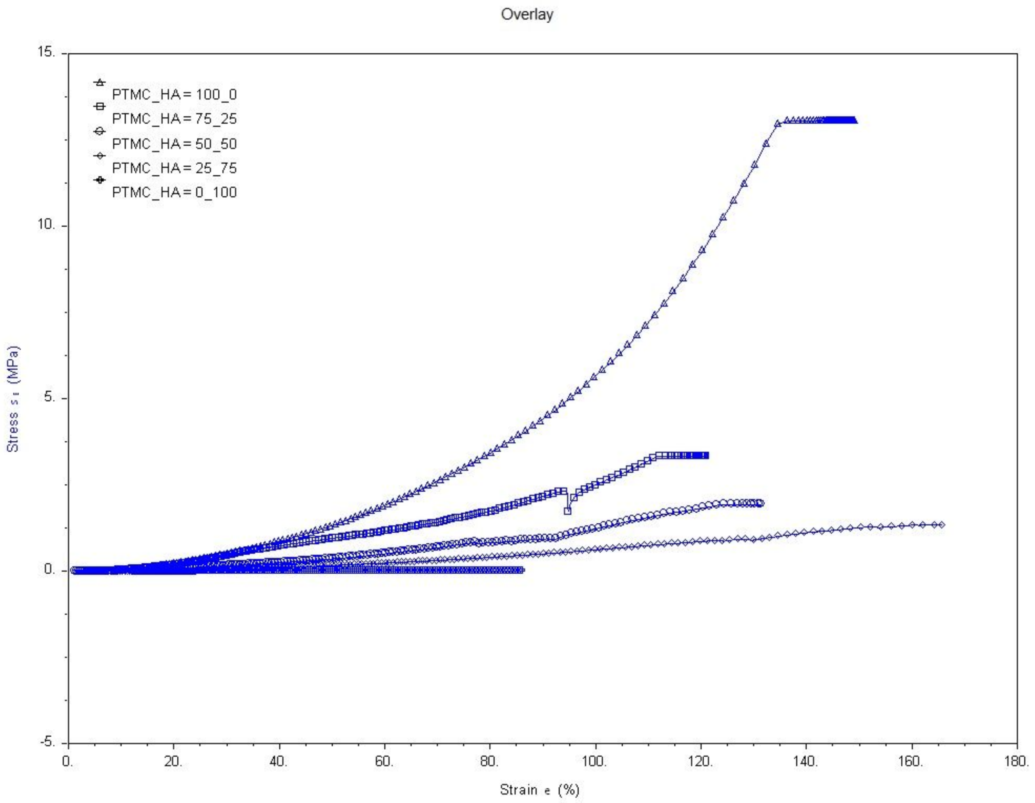

Figure S4. Compression stress-strain curves of hydrated PTMC-tMA:HAMA networks.

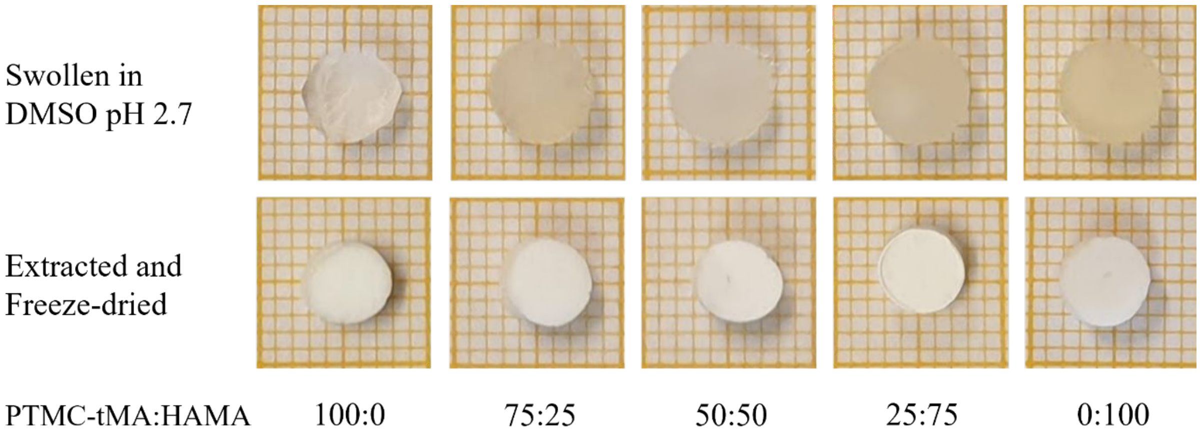

Figure S5. Macroscopic appearance of porous PTMC-tMA:HAMA networks swollen in DMSO pH 2.7 after crosslinking and after subsequent extraction and freeze drying. Small squares are 1x1 mm. The PTMC-tMA:HAMA mixtures were frozen at -25 ^{o}C before and during photo-crosslinking.

References

1. Vasi, A. M.; Popa, M. I.; Butnaru, M.; Dodi, G.; Verestiuc, L. Chemical functionalization of hyaluronic acid for drug delivery applications. *Mater. Sci. Eng., C* **2014**, *38*, 177–185.

2. Zhang, F.; He, C.; Cao, L.; Feng, W.; Wang, H.; Mo, X.; Wang, J. Fabrication of gelatin–hyaluronic acid hybrid scaffolds with tunable porous structures for soft tissue engineering. *Int. J. Biol. Macromol.* **2011**, *48*, 474–481.

3. Guo, Z.; Liang, J.; Ankone, M. J. K.; Poot, A. A.; Grijpma, D. W.; Chen, H. Fabrication of poly (trimethylene carbonate)/reduced graphene oxide-graft-poly (trimethylene carbonate) composite scaffolds for nerve regeneration. *Biomed. Mater. (Bristol, U. K.)* **2019**, *14*, 024104.

4. Liu, X.; Liu, S.; Li, K.; Feng, S.; Fan, Y.; Peng, L.; Wang, X.; Chen, D.; Xiong, C.; Bai, W.; Zhang, L. Preparation and degradation characteristics of biodegradable elastic poly (1,3-trimethylene carbonate) network. *Polym. Degrad. Stab.* **2021**, *193*, 109718.

5. Truong, V.; Blakey, I.; Whittaker, A. K. Hydrophilic and amphiphilic polyethylene glycol-based hydrogels with tunable degradability prepared by "click" chemistry. *Biomacromolecules* **2012**, *13*, 4012–4021.
